# Incidence and risk factors of neonatal bacterial infections: a community-based cohort from Madagascar (2018–2021)

**DOI:** 10.1186/s12879-023-08642-w

**Published:** 2023-10-05

**Authors:** Ines Devred, Lison Rambliere, Perlinot Herindrainy, Lovarivelo Andriamarohasina, Aina Harimanana, Frederique Randrianirina, Elisoa Hariniaina Ratsima, Delphine Hivernaud, Elsa Kermorvant-Duchemin, Zafitsara Zo Andrianirina, Armya Youssouf Abdou, Elisabeth Delarocque-Astagneau, Didier Guillemot, Tania Crucitti, Jean-Marc Collard, Bich-Tram Huynh

**Affiliations:** 1 CESP, Anti-infective evasion and pharmacoepidemiology team, Université Paris-Saclay, UVSQ, Inserm, Montigny-Le-Bretonneux, F- 78180 France; 2 Institut Pasteur, Université Paris Cité, Epidemiology and Modelling of Antibiotic Evasion (EMAE), Paris, F-75015 France; 3 Epidemiology Unit, Institut Pasteur de Madagascar, Antananarivo, Madagascar; 4 Centre de biologie clinique, Institut Pasteur de Madagascar, Antananarivo, Madagascar; 5 Hôpital Necker-Enfants malades, Department of Neonatal medicine, AP-HP, Université Paris Cité, Paris, France; 6 Peadiatric Ward, Centre Hospitalier de Soavinandriana, Antananarivo, Madagascar; 7 Medical Information, AP-HP. Paris Saclay, Public Health, Clinical research, Le Kremlin-Bicêtre, F-94276 France; 8 Experimental Bacteriology Unit, Institut Pasteur de Madagascar, Antananarivo, Madagascar

**Keywords:** Neonatal, Severe Bacterial Infection, Antibiotic resistance, Madagascar

## Abstract

**Background:**

Few studies on neonatal severe bacterial infection are available in LMICs. Data are needed in these countries to prioritize interventions and decrease neonatal infections which are a primary cause of neonatal mortality. The BIRDY project (Bacterial Infections and Antimicrobial Drug Resistant among Young Children) was initially conducted in Madagascar, Senegal and Cambodia (BIRDY 1, 2012–2018), and continued in Madagascar only (BIRDY 2, 2018–2021). We present here the BIRDY 2 project whose objectives were (1) to estimate the incidence of neonatal severe bacterial infections and compare these findings with those obtained in BIRDY 1, (2) to identify determinants associated with severe bacterial infection and (3) to specify the antibiotic resistance pattern of bacteria in newborns.

**Methods:**

The BIRDY 2 study was a prospective community-based mother and child cohort, both in urban and semi-rural areas. All pregnant women in the study areas were identified and enrolled. Their newborns were actively and passively followed-up from birth to 3 months. Data on clinical symptoms developed by the children and laboratory results of all clinical samples investigated were collected. A Cox proportional hazards model was performed to identify risk factors associated with possible severe bacterial infection.

**Findings:**

A total of 53 possible severe bacterial infection and 6 confirmed severe bacterial infection episodes were identified among the 511 neonates followed-up, with more than half occurring in the first 3 days. For the first month period, the incidence of confirmed severe bacterial infection was 11.7 per 1,000 live births indicating a 1.3 -fold decrease compared to BIRDY 1 in Madagascar (p = 0.50) and the incidence of possible severe bacterial infection was 76.3, indicating a 2.6-fold decrease compared to BIRDY 1 in Madagascar (p < 0.001). The 6 severe bacterial infection confirmed by blood culture included 5 *Enterobacterales* and one *Enterococcus faecium*. The 5 *Enterobacterales* were extended-spectrum β-lactamases (ESBL) producers and were resistant to quinolones and gentamicin. *Enterococcus faecium* was sensitive to vancomycin but resistant to amoxicillin and to gentamicin. These pathogns were classified as multidrug-resistant bacteria and were resistant to antibiotics recommended in WHO guidelines for neonatal sepsis. However, they remained susceptible to carbapenem. Fetid amniotic fluid, need for resuscitation at birth and low birth weight were associated with early onset possible severe bacterial infection.

**Conclusion:**

Our results suggest that the incidence of severe bacterial infection is still high in the community of Madagascar, even if it seems lower when compared to BIRDY 1 estimates, and that existing neonatal sepsis treatment guidelines may no longer be appropriate in Madagascar. These results motivate to further strengthen actions for the prevention, early diagnosis and case management during the first 3 days of life.

**Supplementary Information:**

The online version contains supplementary material available at 10.1186/s12879-023-08642-w.

## Background

Neonatal infections remain a major cause of morbidity and mortality in the world, and are involved in more than half of all neonatal deaths [1]. Severe bacterial infections are a leading cause of neonatal infections with great majority occurring in low and middle-income countries (LMICs). In addition in LMICs, multiple factors lead to the emergence and spread of drug-resistant bacteria [2]. These pathogens might become increasingly resistant to multiple drugs. In addition, second line antimicrobial drugs required to treat these resistant infections are not always accessible for people living in these settings.

Considerable geographical differences exist in terms of incidence, bacterial resistance, and mortality of neonatal bacterial infection, which is disproportionately high in sub-Saharan Africa and South Asia [3, 4]. Accurate knowledge of incidence of neonatal severe bacterial infection in LMICs, as well as their determinants and resistance pattern, is essential to implement and monitor health policy interventions, and to guide resources allocation.

The majority of available studies in LMICs have been conducted in hospitals. However, home delivery is not uncommon in LMICs. Infected home-borne neonates might not reach hospital on time, and they may deteriorate very quickly. This might lead to biased estimates of severe bacterial infections incidence, subsequent outcomes, and their etiology. Therefore, investigating neonatal severe bacterial infections in community in LMICs is important, but challenging due to the specificities of the context, such as limited accessibility, resources, cultural variations, data collection constraints, and ethical considerations.

The BIRDY project (Bacterial Infections and Antimicrobial Drug Resistant Diseases among Young Children), whose main objective was to estimate the incidence of neonatal severe bacterial infection in community in LMICs, was conducted in Madagascar, Senegal and Cambodia, specifically to address these challenges [5]. Results of the BIRDY project (2012–2018) showed a particularly high incidence of Severe Bacterial Infection (SBI) in Madagascar (196.3 possible SBI per 1,000 live births [95% CI = 176.5 to 218.2]), nearly twice as high as in Cambodia and Senegal (110.1 [88.3 to 137.3] and 78.3 [59.5 to 103] per 1,000 live birth, respectively) [5]. In addition to its scientific objectives, the BIRDY project also included a large component of capacity building, especially towards health care providers, mothers and traditional birth attendants to improve detection and management of severe bacterial infection.

The second part of the BIRDY project (BIRDY 2) was launched in Madagascar in 2018, and it was an opportunity to take stock of severe neonatal infections and its resistance 10 years after the start of the BIRDY project.

We here (1) estimate the incidence of neonatal severe bacterial infections and compare these findings with those obtained with BIRDY 1 project in Madagascar, (2) identify determinants associated with severe bacterial infection, (3) and specify antibiotic resistance pattern of bacteria.

## Methods

### Study design

The BIRDY project was a prospective multicentric community-based mother and child cohort. The BIRDY study was initially conducted between 2012 and 2018 in Madagascar, Senegal and Cambodia in both urban and rural areas (BIRDY 1). The project continued in Madagascar only between September 2018 and September 2021. The methodology of the BIRDY project has already been described in detail elsewhere [5] and the neonatal follow-up was similar between BIRDY 1 and BIRDY 2.

### Study area and recruitment

Study population included all neonates born in 3 neighborhoods (Avaradoha, Besarety, and Soavinadriana) of Antananarivo (the capital of Madagascar, with a catchment area population of 14 997) and those of the rural city of Moramanga (catchment area population of 17 159). All pregnant women in the study areas were identified with help of the community healthcare workers in order to exhaustively include all live births at delivery. This methodology allowed us to enroll neonates born at home, who would have been missed in case of neonates recruitment directly at delivery in health-care facilities. Inclusion criteria for pregnant women included usual residence in the study area with no intention of moving during the follow-up period, no objection to the ongoing research or to the collection of biological samples and having given written informed consent. Whereas in BIRDY 1 pregnant women were included during the third trimester of pregnancy, they were included from the first trimester of pregnancy in BIRDY 2.

Once enrolled, one antenatal visit was planned monthly and pregnant women were regularly contacted around the due date, so that the investigation team could be present as close as possible to the delivery (within 24 h). At delivery, neonates inclusion criteria were live newborns born to parent living in the study area with no intention of moving during the follow-up period, and whose parents were informed and consented to the ongoing research. To ensure the exhaustiveness of live-birth recruitment, all newborns meeting the inclusion criteria could be included directly at birth at the health care facility, even if their mothers had not been enrolled during their pregnancy.

### Newborns follow-up: community based surveillance

At birth, newborns presenting a risk factor for infection, including premature rupture of membrane (> 24 h), fetid amniotic fluid, dystocic delivery, antibiotic therapy or maternal fever at delivery, were examined by a physician. Then, during the first three months of life, newborns were actively and passively monitored. Home visits made by study investigator were planned as part of the active monitoring: two visits were scheduled during the first week of life, then one visit per week until the end of the first month, and then two visits per month until the third month. Active follow-up ended-up at 3 months. These routine check-ups were conducted in order to minimize number of missed suspected infection and to obtain anthropometric measurements. Passive monitoring consisted of surveillance of the infant by the mother, who was asked to contact a study investigator if the infant showed signs of infection. For this purpose, mothers were taught the main signs of infection and how to take the infant’s temperature. After 3 months, 3 visits were planned at 6, 9 and 12 months to evaluate child’s growth and neurocognitive development.

### Assessment and management

During follow-up, in presence of infection criteria, neonates were referred to a study collaborating physician or directly at the referring hospital in presence of signs of severity. If severe infection was suspected, blood tests and culture were systematically drawn and others samples (such as urine analysis, stool culture, lumbar puncture) performed as defined by an algorithm which was based on the WHO recommendations [6]. Antimicrobial drugs were empirically administered according to the WHO and at discretion of the physician. Bacteriological samples were taken as much as the possible before first antibiotic administration. Bacteriological results were reported to the physicians. Decisions regarding patient care and antimicrobial drug treatments were left to the physicians to decide according to local protocols.

### Sampling and bacteriologic analyses

Biological samples were taken by healthcare team who collaborated with the project and who took care of the child in case of illness. If a severe bacterial infection was suspected, a venous blood culture was taken, with the volume of blood collected based on child’s weight. Amount collected varied from 0.5 ml for children under 1 kg to 2 ml for those weighing up to 6 kg. In cases of diarrhea, a sterile container was used to collect a stool sample equivalent to one tablespoon in quantity, ensuring the integrity of the sample. All collected samples (including blood, stool, urine, and cerebrospinal fluid) were immediately packaged and transported in a secure, cooled container to the microbiological laboratory at the Institut Pasteur in Madagascar to ensure rapid delivery.

Blood samples were incubated using the BACTEC automated system for up to 5 days at a temperature of 35 ± 2 °C. If positive, a direct microscopic examination of broth and gram staining were performed. Fresh blood agar and chocolate media were used for bacterial isolation, and bacterial strains were identified using matrix-assisted laser desorption/ionization time-of-flight (MALDI-TOF) mass spectrometry. Antimicrobial susceptibility testing was done by disk diffusion. The methods recommended by the French Society for Microbiology (FSM) were used for bacterial isolation, identification, and the corresponding breakpoint guidelines were followed to interpret results of antimicrobial susceptibility testing. All Enterobacterales that were resistant to third-generation cephalosporin underwent a double-disk synergy test to detect ESBL producers. This test was performed systematically and followed guidelines established by FSM. Escherichia coli ATCC 25,922 was used for quality control strains.

Urine samples were examined macroscopically and microscopically, followed by leukocyte count and Gram staining. Cultures were inoculated on selective media and incubated for 24–48 h at 35 ± 2 °C. Isolated colonies were identified with MALDI-TOF. CSF samples were visually classified and counted for red and white blood cells. Quantitative cytology was performed after cytocentrifugation and gram staining. Specific pathogens were targeted for soluble antigen detection. Two drops of CSF were inoculated on agar and incubated for 5 days. Isolated colonies were identified with MALDI-TOF. All susceptibility testing were done according to the FSM guidelines.

### Data collection

Study investigators were responsible for collecting epidemiological data. During pregnancy, study investigators collected socioeconomic data and maternal information (medical and gynaecological history, treatment, obstetrical follow-up). Information on labour and delivery were also collected, including risk factors for infection, as well as data on first neonatal assessment: vitality score (APGAR score), anthropometric data (weight, height, head circumference), neonatal resuscitation, if any.

At every home visit, study investigators recorded anthropometric data (weight, height, cephalic and brachial circumference) and presence of any signs of severe infection in the infant. Medical assessments carried out during follow-up, if any, were also collected, including symptoms and clinical signs, treatments carried out, and any diagnosis retained. Finally, data on any deaths or lost to follow-up were recorded.

The end of the BIRDY 2 study period (March to August 2020) overlapped with Covid-19 epidemic in Madagascar (first case of Covid 19 detected in March 2020 in Madagascar), while 51 neonates were born and followed during this period. In Madagascar, restrictive measures (including national lockdown), which were adapted according to the evolution of the situation, were implemented between March to October 2020. Internal procedures have been put in place to ensure safety of the mothers, their newborns and the investigation team while adapting the research protocol to reach the scientific objectives of the project: active phone monitoring by investigation team for example.

### Sample size

Previous findings in the same study area revealed an incidence of confirmed neonatal infections of 15.2 [10.6–21.8] per 1,000 live births [5]. Based on these prior findings, a sample size of 600 newborns would provide an estimate of the incidence of neonatal infections with a precision of approximately ± 2%, assuming a 95% confidence level. This sample size is appropriate for estimating the incidence of neonatal infections in the target population.

### Study definition

We defined possible Severe Bacterial Infection (**pSBI**) episode as presence of at least one sign among the following: chest indrawing, tachypnea (> 60/min), hypo or hyperthermia (axillary temperature < 35 °C or > 37.5 °C, respectively), lethargy, convulsion or poor feeding. Neonates presenting with tachypnea as the only sign were not considered as cases because of low specificity of this sign when isolated [7]. All cases were reviewed by a committee composed of a medical epidemiologist, a neonatologist and an infectious disease specialist, to classify them and exclude non-severe cases and differential diagnoses. For example, post-vaccination fever, viral bronchiolitis and gastroenteritis, conjunctivitis and localised omphalitis were not considered as pSBI.

We defined confirmed Severe Bacterial Infection (**cSBI**) episode as the presence of clinical signs of severe infection (as defined above) and a positive culture from normally sterile sites: blood or cerebrospinal fluid or urine. Each case was classified as a cSBI or as not clinically relevant by the same expert committee. In particular, cultures positive for bacteria suggestive of contamination such as coagulase negative staphylococcus isolated in blood culture in a clinical context not suggestive of infection, were not considered as a confirmed SBI.

The neonatal follow-up period was defined from birth to 90 days of life. Early-onset sepsis were defined as those occurring in the first week of life, and late-onset sepsis as those occurring from 7 to 90 days of life [8].

In order to compare our findings with literature with follow-up periods varying from 1 to 3 months, we also considered 1-month (30 days) and 2-month period (60 days) of follow-up.

Multi-drug-resistance (MDR) was defined as acquired non-susceptibility to at least one agent in three or more antimicrobial categories [9].

We defined omphalitis as an umbilical discharge or redness extending to umbilical base, regardless of extent or severity.

### Statistical analysis

We used descriptive statistics (proportions, mean, and SDs) to summarize characteristic of mothers and neonates. We compared difference in proportions and means by using the X_2_ and Student-t-test, respectively. If the validity conditions for those parametric tests were not met, we used non-parametric tests: Fisher’s exact test for categorical variables and Mann-Whitney test for quantitative variables. We estimated the incidence of severe neonatal bacterial infection per 1,000 live births. The incidence rates with 95% confidence interval (95% CI) were calculated according to the criteria of possible and confirmed SBI. We calculated incidence rates of pSBI and cSBI for 30 days 60 days and 90 days of neonatal follow-up. The comparison of the different incidence rates were done using “epiR” package in R. Time to the first pSBI and cSBI were described using Kaplan-Meier survival curves and compared using the log-rank test.

We aimed to identify if low birth weight and factors related to delivery (exposures of primary interest: maternal fever, fetid amniotic fluid, premature rupture of the membranes) were associated to early onset pSBI, as early onset infections represented the majority of the overall burden of neonatal severe infection. We performed a Cox proportional hazards regression model and association were expressed as hazard ratios (HRs) with 95% CI. The hypothesis of proportionality of risks was tested for each variable in the model, using the Schoenfeld residual test. Variables that did not respect the assumption of proportional hazards were defined as stratification variables in the multivariable model. We first performed univariate analysis and, factors associated with the outcome with a p-value less than 0.20 in univariate analysis were entered in a multivariable Cox model. A manual stepwise backward procedure was carried out to identify factors independently associated with the outcome. Site (semi-rural or urban) and sex of newborn were forced in the final multivariate model.

Missing data were rare (< 1%), except for the variable “fetid amniotic fluid” with 26 missing data (5%). Newborns with missing data were excluded from the multivariate Cox model and we conducted complete case analyses [10, 11]. Then, we also performed sensitivity analyses, by considering that all missing data for the variable “fetid amniotic fluid”, corresponded firstly to non-fetid amniotic fluid and secondly to the opposite situation.

Some variables were correlated, such as difficult delivery and need for resuscitation at birth or such as primigravidae and maternal age. We included in the multivariate Cox model need for resuscitation at birth because this variable was the most strongly associated with neonatal infection; and maternal age because primigravidae does not respect the assumption of proportional hazards.

In a second step, to document strength and direction between the outcome and variables that did not respect the proportional hazards assumption, a parametric model was carried out with an accelerated failure time (AFT) approach, using a log-normal distribution. The overall analysis strategy was the same as described above for the Cox model.

All p-values were from 2-sided test, and results were deemed statistically significant at p < 0.05. All analysis were conducted with R version 4.1.3.

### Ethical statement

The study was approved by the ethics committees of Madagascar (n° 119 MSANP/CERBM) and the Institutional Review Board of Institut Pasteur (n° IRB/2018/05), France. Written informed consent was obtained for all parents of newborn. If a parent could not read, a witness present during the participant’s information session also countersigned the consent form to confirm the collection of the participant’s informed consent.

## Results

**Fig. 1:**
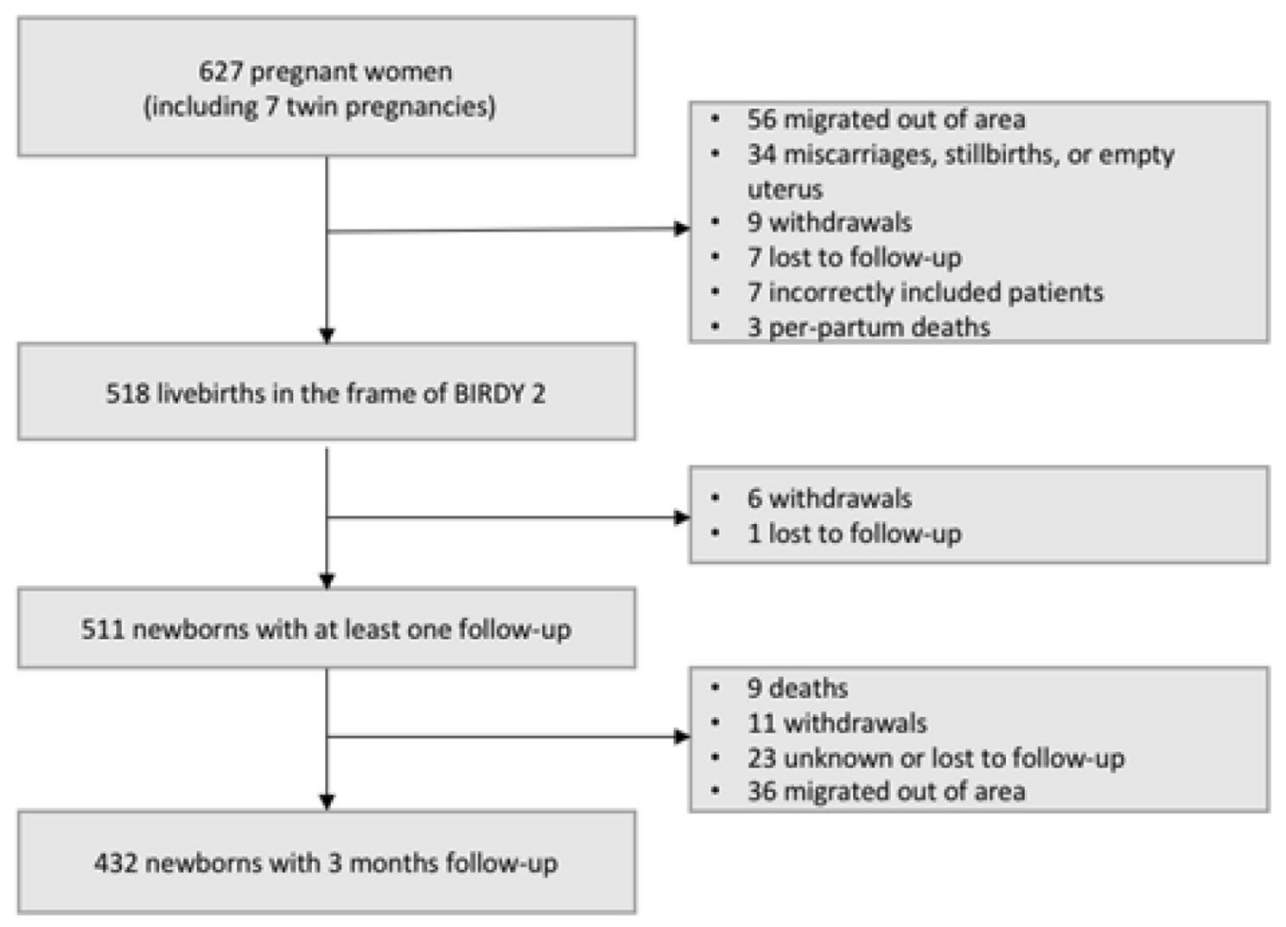
Study flow diagram

A total of 627 pregnant women were enrolled in the study, of which 309 were from semi-rural area and 318 from urban area. Of these pregnancies, 518 live newborns were included at delivery: 511 newborns had at least one medical evaluation during the follow-up period and were considered as our study population. We noted 6 withdrawals of consent immediately after the birth. There were 260 (50.9%) boys and 251 (49.1%) girls, 46 (9%) were low birth weight newborns (< 2500 g). The majority of deliveries (60.9%) took place in a health facility or at the midwife’s office. There were 71 (13.9%) caesarean sections, 66 (12.9%) dystocic deliveries, and 68 (13.3%) neonatal resuscitations. Characteristics of mothers and newborns by site are presented in Table 1. Overall, 432 (84.5%) newborns were followed up for 3 months (Fig. 1).

**Table 1:** Characteristics of mothers and their neonates

	Total	Urban (Antananarivo)	Semi-rural (Moramanga)	p-value
**Mothers**				
N	**627**	**318**	**309**	
**Age** (years): median [min-max]	23.7 [14–44]	23.6 [14–44]	23.8 [14–44]	0.51
**Education**				0.33
Absence or primary school	123 (19.6%)	62 (19.5%)	61 (19.7%)	
Partial secondary school	345 (55.0%)	183 (57.5%)	162 (52.4%)	
Complete secondary or higher	159 (25.4%)	73(23.0%)	86 (27.8%)	
**Household members**				0.53
Less than 4 people	334 (53.3%)	166 (52.2%)	168 (54.4%)	
Between 4 and 8 people	265 (42.3%)	135 (42.5%)	130 (42.1%)	
8 people or more	28 (4.5%)	17 (5.3%)	11 (3.6%)	
**Housing with electricity**	490 (78.1%)	275 (86.5%)	215 (69.6%)	< 0.001
**Latrine**				< 0.001
No latrine	24 (3.8%)	23 (7.2%)	1 (0.3%)	
Outdoor latrine	559 (89.2%)	277 (87.1%)	282 (91.3%)	
Indoor latrine	44 (7.0%)	18 (5.7%)	26 (8.4%)	
**Water treatment**				0.007
No water treatment	186 (29.7%)	106 (33.3%)	80 (25.9%)	
Boiled water	384 (61.2%)	188 (59.1%)	196 (63.4%)	
Alternative water treatment	71 (11.3%)	25 (7.9%)	46 (14.9%)	
**Primigravidae**	210 (33.5%)	103 (32.4%)	107 (34.6%)	0.55
**Poor nutritional status** ^1^	22 (3.51%)	16 (5.03%)	6 (1.94%)	0.04
**Miscarriage or stillbirth**	34 (5.4%)	20 (6.3%)	14 (4.5%)	0.33
**Previous antenatal visit at enrollment**	612 (97.6%)	311 (97.8%)	301 (97.4%)	0.75
**Newborns and delivery**				
N	**511**	**259**	**252**	
**Boy**	260 (50.8%)	125 (48.3%)	135 (53.6%)	0.23
**Low Birth weight** (< 2500 g)	46 (9.0%)	28 (10.9%)	18 (7.1%)	0.14
**Birthweight** (grams): median [min-max]	3000 [1100–4500]	2900 [1160–4000]	3100 [1100–4500]	< 0.001
**Birth attendant**				< 0.001
Midwife	292 (57.3%)	143 (55.4%)	149 (59.1%)	
Physician	77 (15.1%)	61 (23.6%)	16 (6.3%)	
Nurse	25 (4.9%)	2 (0.8%)	23 (9.1%)	
Traditional birth attendant	116 (22.7%)	52 (20.2%)	64 (25.4%)	
**Delivery at home**	200 (39.1%)	85 (32.8%)	115 (45.6%)	0.003
**Cesarean section**	71 (13.9%)	45 (17.4%)	26 (10.3%)	0.02
**Premature rupture of the membranes > 24 h**	14 (2.9%)	4 (1.7%)	10 (4.0%)	0.11
**Fetid amniotic fluid**	31 (6.4%)	14 (5.9%)	17 (6.9%)	0.64
**Difficult delivery**	66 (13.5%)	28 (11.6%)	38 (15.3%)	0.23
**Resuscitation at birth**	68 (13.3%)	30 (11.6%)	38 (15.1%)	0.25

Estimates are calculated based on non-missing data

^1^ Poor nutritional status in mothers was defined as mid-upper-arm circumference < 21 cm

### Incidence of severe bacterial neonatal infections

During the total follow-up period of 3 months, 6 out of 511 neonates were classified as having cSBI (n = 4 in urban area and n = 2 in semi-rural area) and all were cases of bacteraemia (Annex, S1). The overall incidence of cSBI was 11.7 per 1,000 live births [95% CI = 4.3–25.6]. These confirmed cases occurred mainly in the first week of life: 4/6 occurred during the first 3 days of life, 5/6 occurred during the first week, and the sixth infection occurred on the 22nd day of life. A total of 53 out of 511 neonates were classified as having possible SBI, resulting an overall incidence of 103.7 per 1,000 live births [95% CI = 77.7-135.7]. The overall incidence of pSBI was 127.0 [95% CI = 86.9-179.3] per 1,000 live births (n = 32) in the semi-rural area, and 81.1 [95% CI = 50.2-123.9] per 1,000 live births (n = 21) in the urban area, p = 0.09. The majority of these pSBI (32/53, 60.4%) cases also occurred in the first week of life: the incidence of early onset pSBI was 62.6 per 1,000 live births [95% CI = 42.8–88.4], whereas the incidence of late onset pSBI was 44.5 per 1,000 live births [95% CI = 27.54–68.01], which is equivalent to an increase in incidence density rate of roughly 16.5 times for early-onset pSBI when compared to late-onset pSBI.

**Fig. 2:**
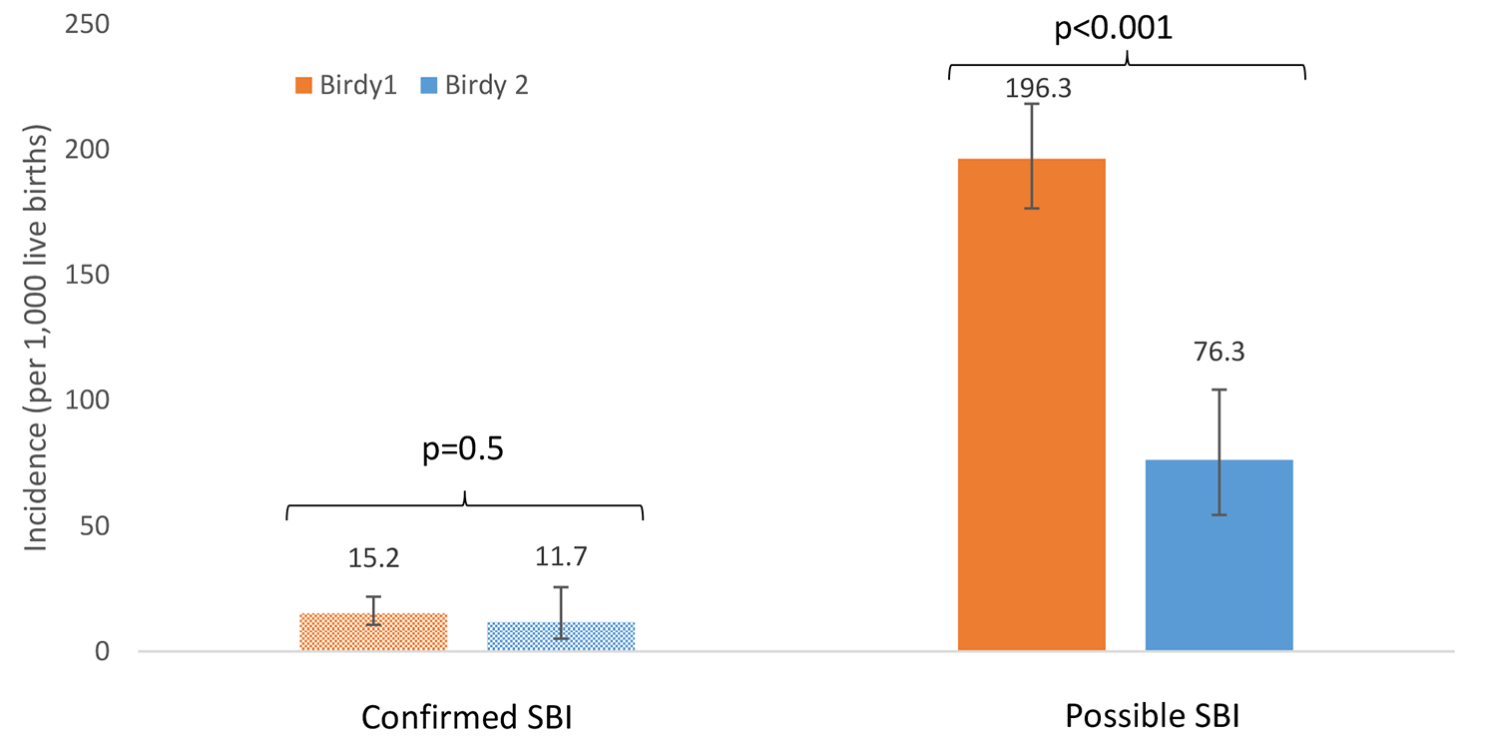
Incidence ratios per 1,000 live births of cSBI and pSBI between BIRDY 1 and BIRDY 2 for the first month of life

If we considered the first month period of follow-up, the incidence of cSBI was 11.7 per 1,000 live births [95% CI = 4.3–25.6], so a 1.3 -fold decrease compared to BIRDY 1 project in Madagascar (15.2 in BIRDY 1, p = 0.50), and the incidence of pSBI was 76.3 [95%CI = 54.3-104.3], so a 2.6-fold decrease compared to BIRDY 1 project in Madagascar (193.3 in BIRDY 1 versus 76.3 in BIRDY 2, p < 0.001). These results are illustrated in Fig. 2.

Considering a 2 month-period of follow-up, the incidence of cSBI was 11.7 per 1,000 live births [95% CI = 4.3–25.6] and the incidence of pSBI was 92.0 [67.6-122.3] per 1,000 live births.

### Clinical characteristics and antibiotics therapy of pSBI and CSBI in the 90 first days of life

#### Clinical characteristics

Among the 53 cases of pSBI, 15 infants (28.3%) were low birth weight, 17 (32.1%) were preterm (less than 37 weeks gestational age), 9 (17%) infants were born in a context of fetid amniotic fluid and 22 (41.5%) were resuscitated at delivery. The most common clinical signs encountered in pSBI were poor feeding (43.4%), fever (41.5%), drowsiness/hypotonia (32.1%) and severe chest indrawing (30.2%). Diagnosis retained by the attending physician were suspected meningitis (n = 4), pneumonia (n = 18), and sepsis (n = 32), including 3 secondary to omphalitis. Blood cultures were drawn from 34 of the 53 infants (64.2%), and lumbar puncture from 2 to 53 infants. Among infants with a pSBI, 9 died, including 6 in the first week, which results in a case fatality rate of 17.0%.

Among the 6 infants with cSBI, all confirmed with positive blood cultures, half had a birth weight < 2000 g. Final diagnosis retained by the attending physician were meningitis in one case, pneumonia in one case, and 4 septicaemia for the 4 remaining cases. Among infants with cSBI, one died at 22 days of life from a *Klebsiella sp* infection. Clinical features of cSBI are shown in Annex (S1).

#### Antibiotics therapy

Among the 53 pSBI cases, 51 infants received antibiotic treatment. The majority (n = 32, 62.7%) received a third- generation cephalosporin (cefotaxime, ceftriaxone), 27.5% (n = 14) received amoxicillin and 9.8% (n = 5) received amoxicillin/clavulanic acid; 45.1% (n = 23) received gentamicin in addition to a 3rd generation cephalosporin. Only one infant received the WHO-recommended first line of antibiotic therapy in case of suspected neonatal sepsis, i.e. amoxicillin-gentamicin combination therapy. Regarding the 6 cases of cSBI cases, 4 received a combination of 3rd generation cephalosporin and gentamicin, one received amoxicillin alone, and antibiotic data were not available for the last neonate. Among them, one neonate died and the remaining five neonates progressed favorably, with 2 of them being switched to imipenem/cilastatin.

#### Omphalitis

Twenty six cases of omphalitis were diagnosed: 10 (38.5%) were diagnosed within the first 7 days, and 11 (42.3%) occurred in home-born infants. The incidence of omphalitis was 50.9 [95%CI = 33.24–74.55] per 1,000 live births. Among these, umbilical discharge was sampled in eight cases, with 6 positive cultures: *Escherichia coli* (n = 1), *Acinetobacter baumanii* (n = 1), *Enterobacter sp* (n = 1), *Pseudomonas aeruginosa* (n = 1) and coagulase-negative staphylococci (n = 2). Twelve neonates (46.1%) were treated with systemic therapy.

### Pathogens isolated and antibacterial resistance of pSBI and CSBI in the 90 first days of life

Among the 6 cases of cSBI, the main pathogen isolated was *Klebsiella sp* (n = 4, one *Klebsiella pneumonia* one *Klebsiella sp, Klebsiella variicola* in 2 twins), the two other pathogens identified were *Enterobacter asburiae* and *Enterococcus faecium*. The 5 Enterobacterales were extended-spectrum β-lactamases (ESBL) producers, and were resistant to quinolones and gentamicin. They are thus classified as MDR bacteria, but remained susceptible to carbapenem. The five Enterobacterales were resistant to gentamicin in addition to their natural resistance to amoxicillin. *Enterococcus faecium* was sensitive to vancomycin but resistant to amoxicillin and to gentamicin. Therefore, all of these bacteria were resistant to the two antibiotics recommended by the WHO in case of neonatal sepsis.

### Risk factors associated with early onset pSBI

Results of the univariate and multivariate analyses are shown in Table 2.

In the multivariate Cox model, infants born in a context of fetid amniotic fluid and those who had resuscitation at birth were more at risk of early onset pSBI (adjusted HRs: 5.33 [2.24–12.71], p < 0.001 and 7.91 [3.76–16.67], p < 0.001 respectively). Low birth weight did not meet the proportional hazards assumption. This variable was therefore included in the multivariate model as a stratification variable. Twin pregnancy was strongly associated with early pSBI in the univariate model (< 0.001), and the association did not remain significant in the multivariate Cox model.

In the multivariate AFT model, time to onset of pSBI was 25 shorter in neonates with a low birth weight than in those with a normal birth weight (adjusted time ratio = 0.04 [0.01–0.19], p < 0.001), which mean that being low birth weight sped-up time to pSBI. Results of the multivariable Cox model and the AFT model remained the same for the other co-variables.

**Fig. 3:**
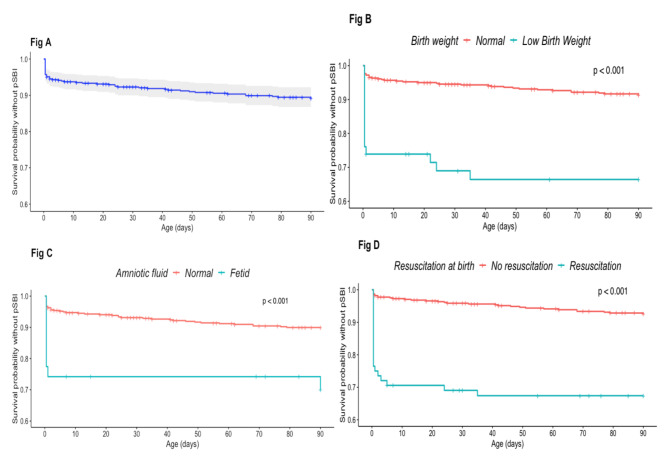
(BIRDY 2, 2018–2021, n = 511). Kaplan Meier curves. Fig **A)** Overall pSBI, Fig **B)** pSBI according to birth weight. Fig **C)** pSBI according to fetid amniotic fluid status at delivery. Fig **C)** pSBI according to need for resuscitation requirement at birth

Overall incidence of pSBI and according to birth weight, fetid amniotic fluid at delivery and need for resuscitation at birth are represented by a Kaplan Meier curve over a 3-month period (Fig. 3).

Complete case and sensitivity analyses yielded comparable results.

**Table 2:** Risk factors analysis of possible severe bacterial neonatal infection (BIRDY 2, 2018–2021)

Variables (n = 511)	Univariate cox model		Multivariate cox model		Multivariate AFT model	
	Crude HR	*P*	Adjusted HR	*P*	Adjusted time ratio	*P*
**Girl (boys as reference)**	0.81 [0.4–1.62]	0.54	0.75 [0.36–1.55]	0.43	1.43 [0.45–4.55]	0.54
**Semi-rural area (urban site as reference)**	1.50 [0.75–3.06]	0.25	1.79 [0.77–4.17]	0.17	0.35 [0.09–4.55]	0.11
**Mother’s age > 24 (ref: under 24)**_**1**_	0.48 [0.23–0.99]	0.048				
**Education**						
Absence or primary school	Ref					
Partial secondary school	1.25 [0.47–3.35]	0.65				
Complete secondary school or higher	1.15 [0.38–3.53]	0.80				
**Household without electricity**	0.80 [0.33–1.94]	0.62				
**Poor maternal nutritionnal status**_**2**_	1.90 [0.45–7.94]	0.38				
**Twins pregnancy**	9.23 [3.55–23.99]	< 0.001	2.32 [0.67-8.00]	0.18	0.17 [0.02–1.73]	0.13
**Delivery outside a health facility**	0.50 [0.23–1.12]	0.09				
**Birth attendant**						
Midwife or physician	Ref					
Nurse	0.56 [0.08–4.14]	0.57				
Traditional birth attendant	0.48 [0.17–1.37]	0.17				
**Caesarean section**	2.11 [0.95–4.71]	0.07				
**Rupture of membranes < 24 H**	2.43 [0.58–10.18]	0.22				
**Fetid amniotic fluid**	5.65 [2.54–12.58]	< 0.001	5.33 [2.24–12.71]	< 0.001	0.06 [0.01–0.28]	< 0.001
**Difficult or dystocic delivery**	6.64 [3.28–13.43]	< 0.001				
**Resuscitation at birth**	12.83 [6.27–26.26]	< 0.001	7.91 [3.76–16.67]	< 0.001	0.03 [0.01–0.11]	< 0.001
**Low birth weight (< 2500 g)**_**3**_			Stratified variable		0.04 [0.01–0.19]	< 0.001

^1^24 years old was the median age. ^2^Poor maternal nutritional status was defined as mid-upper-arm circumference <  21 cm. ^3^Low birth weight did not meet the proportional hazards assumption, therefore was stratified in the multivariate model. In the AFT model, low birth weight was included as an independent covariable

## Discussion

The results of this community-based prospective cohort in Madagascar between 2018 and 2021 showed a high incidence of neonatal infection. In the global context, the incidence rate is significantly higher than that observed in high-income countries and in community settings in LMICs. However, this incidence seems lower when compared to BIRDY 1 estimates: 1.3 and 2.6-fold decrease for cSBI and pSBI respectively (although not significant for cSBI). The most critical period was the first 3 days with more than half of pSBI and cSBI occurring during this period. Results also showed that all 6 cases of cSBI were due to MDR bacteria and were resistant to current WHO neonatal sepsis treatment guidelines.

The neonatal recruitment and follow-up were conducted between December 2018 and August 2020 in BIRDY 2, and between September 2012 and December 2016 in BIRDY 1. The lower incidence rates in BIRDY 2 might reflect a real decrease, as the study population and areas, the seasonality and pattern of infectious diseases remained similar between BIRDY 1 and BIRDY 2 period. Despite necessary monitoring adaptations due to COVID-19 pandemic, the 2 studies remain comparable. Active surveillance continued through telephone communication, and healthcare facilities remained accessible for families seeking assistance for their newborns. COVID-19 epidemic in Madagascar coincided with the final phase of the BIRDY 2 study from March to August 2020, with a limited number of newborns (51) being followed, minimizing potential result influence. Furthermore, among these newborns, those born after March 1, 2020, received the same number of medical assessments as those born before March 2020 (p = 0.77). The possible decrease in incidence observed in BIRDY 2 can be attributed to multiple factors. One factor is reinforcement of preventive measures, such as handwashing by birth attendants and families, during COVID-19 epidemic. These measures may have contributed to a reduction in neonatal infections. However, impact of these measures was likely minimal and cannot solely explain the decrease in incidence as COVID-19 epidemic in Madagascar only coincided with the very end of the BIRDY 2 study period, during which a limited number of neonates were born and followed. Additionally, reduction in neonatal infections observed may also be attributed, in part, to the capacity building activities carried out as part of the BIRDY project, which began 10 years ago. These activities focused on training healthcare workers on hand hygiene and educating traditional birth attendants and mothers on safe delivery practices and the importance of maintaining a clean environment. Improved basic hygiene practices among healthcare workers, traditional birth attendants, and mothers may have contributed to the observed reduction in neonatal infections. Although these results are promising, they should be validated through long-term surveillance and clinical trials evaluating interventions focused on capacity building to prevent neonatal infections.

Importantly, these estimates remain high when compared to those in high income countries. For instance, the incidence of early onset cSBI is ten times higher when compared to those in North America (0.17 to 1 per 1,000 live births) [12–14]. In LMICs, recent studies are scare, but available ones from Asia and Sub-Saharan Africa showed slightly lower incidence of cSBI when compared to results of BIRDY 2 (estimated incidence in Bangladesh (one month follow-up): 3.0, in India (two months follow-up): 6.7 and in Kenya (two months of follow-up): 5.5 cSBI per 1,000 live births) [15–17]. Our results are also higher than those of the ANISA study, a recent international cohort conducted in Bangladesh, India and Pakistan (two months of follow-up), where the incidence of cSBI was estimated at 1.6 per 1,000 live births. All these results seem to confirm that the burden of confirmed neonatal SBI is still high in Madagascar, one of the poorest countries in the world.

We used the same case definition for pSBI and quasi-similar study design as those used in ANISA, which renders comparison between the 2 studies appropriate. We found a similar incidence rate of pSBI considering the 60 day-period (92.0 in BIRDY 2 versus 95.4 in ANISA, per 1,000 live birth). Conversely, a meta-analysis from LMICs (Sub-Sahara Africa, Asia and South America) including about 250 000 neonates found a slightly lower estimate of pSBI incidence (62 per 1,000 live births) in Sub-Saharan Africa. It is worth noting that the majority of studies from Sub-Saharan Africa in this meta-analysis did not include severe chest indrawing as a sign of pSBI, which may therefore explain such differences in pSBI incidences [18].

We found that the first week of life is a particularly high-risk period for severe neonatal infection, and especially the first 3 days, with more than half of SBI occurring in the first 3 days. Our results are consistent with those of the BIRDY 1 cohort and others results in literature [7]. Early onset infections are mainly caused by pathogens acquired before or during delivery and usually represent vertical mother to infant transmission, whereas late onset infection are attributed to organisms acquired from interaction with environment.

In view of the importance of early-onset infection, intervention should target this crucial period and, in this purpose, we identified risk factors associated with early pSBI. Low birth weight, fetid amniotic fluid at delivery and need for resuscitation at birth were the three risk factors strongly associated with early onset pSBI. These risk factors are all clearly identified in the literature [19]. The most important neonatal factor predisposing to infection is prematurity [20]. In our analysis, we considered low birth weight as a proxy for prematurity, as birth weight is a more accessible measure in LMIC, whereas ultrasound (considered as the gold standard for the estimation of gestational age) is not widely available. Preterm infants have a 3–10 times higher incidence of infection than full-term neonates, because of a number of factors, such as their immature immune system [21]. Concerning fetid amniotic fluid at birth, it can be a sign of chorioamniotitis, which might be the origin of an infection in neonate [22]. Finally, need for resuscitation at birth was also identified as a risk factor of pSBI in our cohort and in literature [19, 23]. Infection may hamper adaptation to extra-uterine life, but manual procedures may also favour acquisition of pathogens during resuscitation. Our results suggest that possible integrated interventions to decrease neonatal mortality can be based on (1) education of traditional birth attendants and healthcare workers’capacity building for identification of at risk-newborns, (2) education of the mothers to monitor signs of infection in at-risk newborns to consult at first signs of infection especially during the first week of life, and (3) re-inforcement of pregnant women follow-up during their pregnancy to decrease premature membrane rupture and/or delivery.

In this study, *Enterobacterales* were the most common cause of confirmed infection, with *Klebsiella sp* in 4 out 5 cases, and no positive blood culture for group B streptococcus, which corresponds to findings in several LMICs [2, 5, 23, 24]. These 5 *Enterobacterales* were ESBL producers, and resistant to amoxicillin and gentamicin, which are first line of antibiotic recommended by WHO in case of neonatal sepsis. These pathogens remained sensitive to carbapenems, but these broad-spectrum antibiotics are scarcely available in LMICs. In view of the small-scale results (6 blood cultures), we cannot generalise too hastily and further studies are needed to confirm them. Nevertheless, these results may reflect a worrisome reality in terms of AMR spread in the community in Madagascar and that antibiotic therapy recommended by the WHO is perhaps no longer adapted in this country. In contrast, the large-scale community-based ANISA cohort, showed that 85 (83%) of 102 blood culture isolates were susceptible to penicillin, ampicillin, gentamicin, or a combination of these drugs [7]. However, resistance rates probably vary greatly depending on region of the world and local epidemiology. Some antibiotics are currently being evaluated to treat these drug-resistant infections in neonates [25]. It is important that future implementation of these new broad antibiotics take into account possible geographic heterogeneity of resistance in LMICs.

Of note, the 6 neonates with cSBI were initially treated with antibiotics that were not sensitive according to the antibiogram. Among them, one neonate died quickly, 2 others were switched to imipenem/cilastatin and progressed favorably. Three of them showed improvement with antibiotics that were not sensitive according to antibiogram. Although antibiotic resistance is often associated with therapeutic failure, it is not always the case as there may still be some level of effectiveness, albeit with lower probability of success. Moreover, it is possible that for these 3 neonates, initial bacterial load was relatively low and therefore sufficiently reduced by the antibiotic therapy. A lower bacterial load may also enable the immune system to clear the infection without the need for specific antibiotic treatment.

We acknowledge some limitations. The first limitation is the possible underestimation of cSBI. Indeed, bacterial documentation in neonates is difficult, and all the more in in LMIC. In this study, although it was recommended and accessible as part of the study, blood cultures were not performed in more than one third of newborns with pSBI (versus more than half in BIRDY 1). Blood cultures have poor sensibility and specificity in newborns, because they are difficult to draw in neonates [26], and this is especially true in LMIC, requiring skilled personnel and adequate blood volume. In our study, very few cerebrospinal fluid (2 lumbar punctures) were collected, even when 11 meningitis were suspected, likely because physicians are not used to perform lumbar puncture in routine practice. In addition, there may be an overestimation of pSBI. All pSBI might not be of bacterial origin. In addition, clinical signs of infection are often subtle and non-specific in the neonatal period and overlap with non-infectious events. Therefore, we cannot exclude that some pSBI may capture other differential diagnoses such as complication of preterm birth, and perinatal hypoxia-ischemia. However, this definition for “pSBI” is also adopted by other studies and, although it favours sensibility rather than specificity, it allows comparisons between different studies [7, 18]. Given methodological challenges associated with estimating incidence of SBI in LMICs, we endeavored to provide estimates for both pSBI and cSBI, while acknowledging that the true incidence of SBI is likely to fall within the range of these two estimates.

## Conclusion

This community-based prospective cohort provides unique data from Madagascar. It highlights that there is still a high incidence of neonatal infection in this country (2012–2018), even if this incidence seems lower when compared to BIRDY 1 (2018–2021) estimates for the first month of life. These results are encouraging and motivate to further strengthen actions against neonatal infections, which are a preventable and curable cause of neonatal death. Our findings suggest possible avenues of research and intervention to decrease the burden of neonatal severe bacterial infection and consequently might help to achieve Sustainable Development Goal 3 which aims to reduce neonatal death to less than 12 per 1,000 live births for each country by 2030 [27].

### Electronic supplementary material

Below is the link to the electronic supplementary material.

Supplementary Material 1

## Data Availability

Data cannot be shared publicly because data privacy is subject to French and Malagasy regulations. Data are available from the institutional review board of Institut Pasteur in Paris, France (contact via irb@pasteur.fr) for researchers who meet the criteria for access to confidential data.
