# Supplementary material for: Incidence and risk factors of neonatal bacterial infections: a community-based cohort from Madagascar (2018–2021)
Source: BMC Infect Dis. 2023 Oct 5;23:658. doi: 10.1186/s12879-023-08642-w (PMC10552278; doi:10.1186/s12879-023-08642-w)
Supplement: Supplementary file 1 — Supplementary Material 1 [file 12879_2023_8642_MOESM1_ESM.docx]

Supplementary

Annex S1: Clinical and bacteriological characteristics of cSBI

|  |  |  |  |  | **At birth** | | | **Antibiotic resistance to** | | |  |
| --- | --- | --- | --- | --- | --- | --- | --- | --- | --- | --- | --- |
|  | **Site** | **Age (days)** | **Sex** | **Sample** | **Place of birth** | **Resuscitation** | **Weight (g)** | **Ampicillin** | **C3G** | **Gentamicin** | **Death** |
| **GRAM +** |  |  |  |  |  |  |  |  |  |  |  |
| Enterococcus Faecium | Urban | 3 | Girl | Blood | Health care | Yes | 2750 | Resistance (R) | R | R | No |
| **GRAM-** |  |  |  |  |  |  |  |  |  |  |  |
| Klebsiella Sp | Urban | 22 | Boy | Blood | Health care | No | 1850 | R | R | R | Yes |
| Klebsiella variicola ^1^ | Urban | 0 | Boy | Blood | Health care | No | 1500 | R | R | R | No |
| Klebisella variicola ^1^ | Urban | 0 | Boy | Blood | Health care | Yes | 1500 | R | R | R | No |
| Enterobacter asburiae | Semi-rural | 1 | Boy | Blood | Health care | No | 3100 | R | R | R | No |
| Klebsiella Pneumoniae | Semi-rural | 6 | Boy | Blood | Home | No | 3350 | R | R | R | No |

^1^ Blood cultures positives to Klebsiella variicola are from two twins
